# Cardiac magnetic resonance predictors of adverse outcomes in Chagas cardiomyopathy

**DOI:** 10.3389/fcvm.2026.1758594

**Published:** 2026-03-17

**Authors:** Nicolás Ariza-Ordóñez, Diego Rangel, Maria Daniela Valderrama-Achury, Antonia Pino Marín, Julián F. Forero, Claudia Jaimes, Carlos Eduardo Guerrero-Chalela, Michael Chetrit, Héctor M. Medina

**Affiliations:** 1 School of Medicine and Health Sciences, Universidad del Rosario, Bogotá, Colombia; 2 Cardiology Department, Fundación Cardioinfantil-Instituto de Cardiología, Bogotá, Colombia; 3 Radiology Department, Fundación Cardioinfantil-Instituto de Cardiología, Bogotá, Colombia; 4 Department of Cardiovascular Medicine, McGill University Health Centre, Montreal, QC, Canada; 5 Pediatric Cardiology and Adult Congenital Heart Disease Institute, Fundación Cardioinfantil-Instituto de Cardiología, Bogotá, Colombia; 6 Department of Cardiology, Baylor Scott and White, Round Rock, TX, United States

**Keywords:** cardiac magnetic resonance, Chagas cardiomyopathy, Chagas disease, heart faiIure, left ventricular dysfunction, late gadolinium enhancement

## Abstract

**Background:**

Chagas cardiomyopathy (CC) is a major cause of cardiac morbidity and mortality in Latin America. The disease presents with varying degrees of myocardial involvement, posing a significant clinical challenge. Multimodal imaging plays a crucial role in patient assessment and management; however, the role of cardiac magnetic resonance (CMR) imaging in this context remains under investigation.

**Objective:**

To evaluate the association between CMR-derived parameters and the occurrence of adverse outcomes in patients with CC.

**Methods:**

Patients with CC underwent comprehensive CMR evaluation using a 1.5-T scanner. Imaging assessments included biventricular volumes, left ventricle ejection fraction (LVEF), right ventricle ejection fraction (RVEF) and late gadolinium enhancement (LGE) for scar analysis. Follow-up data were collected to assess a primary composite outcome comprising all-cause mortality, cardiovascular hospitalization, ischemic stroke, and heart transplantation. All-cause mortality was analyzed as a secondary outcome.

**Results:**

A total of 133 patients were included [median age 64 years, 71 (53.4%) female]. The mean LVEF was 43.3% ± 15%. LV scar was detected in 97% of patients. Myocardial edema, LV aneurysm, and LV thrombus were observed in 21.1%, 21.1%, and 12.8% of patients, respectively. The primary composite outcome occurred in 63 patients (47.4%). In multivariable analysis, age, subendocardial LV scarring, and extensive LV scar (≥6 segments with LGE) were independently associated with the primary outcome. Only age was independently associated with all-cause mortality.

**Conclusions:**

In patients with CC, subendocardial LV scarring and extensive myocardial fibrosis (≥6 segments with LGE) were independently associated with adverse clinical outcomes. These CMR-derived parameters may serve as valuable prognostic indicators in this high-risk population.

## Introduction

Chagas disease is a parasitic infection caused by Trypanosoma cruzi, a primarily vector-transmitted protozoan (1). According to the World Health Organization, an estimated 6–7 million people worldwide are infected with T. cruzi, predominantly in 21 Latin American countries, where it remains an important public health concern (2). Due to migration patterns, an increasing number of cases have recently been detected in non-endemic regions such as the United States and Europe (3, 4).

Chagas cardiomyopathy (CC) is the most common and severe manifestation of chronic Chagas disease, affecting approximately 42% of infected individuals, according to a recent epidemiological meta-analysis (5). The incidence of cardiomyopathy among individuals seropositive for T. cruzi is estimated at 9.2 events per 1,000 person-years (6). Heart failure, arrhythmias, thromboembolic events, and sudden cardiac death are the most common manifestations. Furthermore, CC accounts for most healthcare costs and deaths in this population (1). CC is associated with an estimated annual all-cause mortality rate of 7.9%, with cardiovascular causes accounting for most deaths (7). Compared with other forms of heart disease, CC is associated with nearly double the risk of mortality (8).

Progression from the acute to the chronic phase of Chagas disease can be prevented with antiparasitic treatment; however, this occurs in only approximately 1% of cases, primarily due to delayed diagnosis and limited access to disease-specific therapies (9). Therefore, risk stratification and development of targeted therapeutic strategies remain critical components of disease management. Currently, most clinical recommendations for the assessment and treatment of CC are extrapolated from guidelines for other cardiomyopathies rather than being specific to Chagas disease, highlighting an important knowledge gap (10).

Several markers have been associated with adverse outcomes in CC, including impaired LV systolic function, heart failure, ventricular aneurysms, and ventricular arrhythmias (10, 11). Additionally, risk scores have been validated for predicting adverse outcomes in this population (1, 7, 10–13).

Cardiac magnetic resonance (CMR) imaging has emerged as a complementary tool for the assessment of patients with CC (14). Owing to its unique myocardial tissue characterization and high spatial resolution, CMR provides valuable insights into disease severity. However, its availability remains limited in endemic and rural areas, and implanted cardiac electronic devices—common in patients with CC—may represent a relative contraindication (15).

The purpose of this study was to assess the association between CMR-derived parameters and adverse outcomes in patients with CC, thereby clarifying the prognostic role of CMR in this population.

## Methods

The study protocol was approved by the institutional research ethics committee and was conducted in accordance with the Declaration of Helsinki. All participants provided written informed consent.

### Study design and population

We retrospectively collected data from consecutive adults (aged ≥18 years) with Chagas cardiomyopathy (CC) who underwent cardiac magnetic resonance (CMR) imaging between 2016 and 2022 at a tertiary care center in Colombia (Fundación Cardioinfantil–Instituto de Cardiología, Bogotá, Colombia).

Chagas disease was defined as at least two positive serological test results (enzyme-linked immunosorbent assay, indirect immunofluorescence, or hemagglutination) for Trypanosoma cruzi, or one positive serological test result plus a documented prior diagnosis of Chagas disease. CC was defined as Chagas disease with at least one marker of cardiac involvement, including left ventricular (LV) global or regional systolic dysfunction [defined as LV ejection fraction (LVEF) ≤ 50% or the presence of global or regional wall motion abnormalities on prior echocardiography] or a history of ventricular fibrillation or sustained ventricular tachycardia (10).

Patients with a history of ischemic cardiomyopathy (defined as history of myocardial infarction, coronary revascularization, or evidence of coronary stenosis ≥50% by coronary angiography or computed tomography angiography), diagnosis of other forms of cardiomyopathy (e.g., primary valvular heart diseases, sarcoidosis, amyloidosis, dilated idiopathic or hypertrophic cardiomyopathy), active cancer at the time of inclusion, incomplete information (more than 20% of the studied variables missing), and low-quality CMR images (defined by observers assessment) were excluded.

### Outcomes and follow-up

Baseline demographic, clinical, and echocardiographic data were obtained from medical records. Follow-up data were collected through review of medical records, structured telephone contact (Supplementary S1), and consultation of the national healthcare database [Administradora de los Recursos del Sistema General de Seguridad Social en Salud (ADRES)].

The primary outcome was a composite of all-cause mortality, cardiovascular hospitalization (see Supplementary Data), ischemic stroke, and heart transplantation (16). All-cause mortality was also analyzed as a secondary outcome.

Follow-up time was defined as the interval (in days) from the CMR examination (time 0) to the occurrence of the first event (time-to-first-event analysis). Patients who did not experience an event were censored at the date of the last outpatient visit or telephone contact.

### Cardiac magnetic resonance imaging protocol

CMR studies were performed using a standardized institutional cardiomyopathy protocol on a 1.5-T scanner (Ingenia, Philips Healthcare, Best, The Netherlands) equipped with a 5-element SENSE coil. The protocol included comprehensive volumetric and functional assessment.

Standard multislice two-dimensional cine images were acquired before contrast administration using a balanced steady-state free precession sequence with electrocardiographic (ECG) gating. A stack of 9–12 contiguous short-axis slices covering both ventricles from the mitral annulus to the apex was obtained (slice thickness 8 mm, interslice gap 2 mm).

Myocardial scar was assessed using late gadolinium enhancement (LGE) imaging with a T1-weighted inversion recovery fast gradient echo sequence acquired 10–15 min after intravenous administration of gadobutrol (0.15–0.2 mmol/kg; Gadovist, Bayer AG, Leverkusen, Germany). Images were obtained in short-axis, two-chamber, and four-chamber views with a slice thickness of 10 mm and no interslice gap.

All images were analyzed offline using a dedicated workstation (IntelliSpace Portal 10, Philips Healthcare, Haifa, Israel) by two observers blinded to clinical characteristics and outcomes.

Ventricular volumes and ejection fractions were calculated using Simpson's method from short-axis cine images. End-diastolic and end-systolic volumes of both ventricles were defined as the maximum and minimum cavity volumes during the cardiac cycle, respectively. Papillary muscles were included within the ventricular cavity. Volumes were indexed to body surface area (BSA), calculated using the DuBois and DuBois formula.

Myocardial edema was assessed using a semiquantitative approach. Signal intensity thresholds were defined as ≥2 standard deviations (SD) above the mean signal intensity of remote, visually normal myocardium on T2-weighted short tau inversion recovery (STIR) images. Regions of interest were manually drawn within the myocardium, and skeletal muscle signal intensity was used as an internal reference.

LGE images were acquired using a phase-sensitive inversion recovery (PSIR) sequence. The presence and extent of LGE were assessed using a semiquantitative approach. LGE was classified as transmural when enhancement involved ≥75% of myocardial wall thickness. Non-transmural LGE (<75%) was further categorized as subendocardial or subepicardial based on the predominant location of enhancement.

Scar location and extent were determined according to the 17-segment American Heart Association model (17). All CMR analyses were performed by experienced readers with Level III CMR training or equivalent expertise.

### Statistical analysis

Continuous variables are presented as mean ± standard deviation (SD) when approximately normally distributed, and as median [interquartile range (IQR)] otherwise. Distributional assumptions were evaluated by visual inspection of boxplots and assessment of outliers.

Categorical variables are summarized as counts and percentages. Comparisons between two independent groups were performed using the Student's *t*-test for normally distributed variables with comparable variances (defined pragmatically as a ratio of standard deviations ≤2). When assumptions were not met, the Mann–Whitney *U*-test was used. Categorical variables were compared using the *χ*^2^ test or Fisher's exact test, as appropriate.

Time-to-event analyses for the primary composite outcome were conducted using Cox proportional hazards regression. Time at risk was calculated from the CMR date to the first occurrence of the composite outcome or censoring at last follow-up.

Multivariable model selection was guided by clinical relevance and *a priori* considerations rather than automated or *p*-value–driven procedures. Effect estimates are reported as hazard ratios (HRs) with 95% confidence intervals (CIs). Kaplan–Meier curves were generated for categorical exposures and compared using the log-rank test.

The proportional hazards assumption was evaluated using Schoenfeld residuals (global and covariate-specific tests). Missing data were handled using a complete-case approach (participants with missing covariate data were excluded from the corresponding multivariable model).

To assess multicollinearity and minimize overfitting, variance inflation factors (VIFs) and the condition index were calculated. Highly collinear variables were not included simultaneously, and clinically redundant covariates were excluded to maintain model parsimony relative to the number of observed events.

To evaluate the prognostic role of LV systolic function, survival analyses were stratified according to LVEF categories. The extent of LV scar was defined as the cumulative number of LV segments demonstrating LGE. Exploratory analyses were conducted to identify a clinically meaningful threshold of scar burden using Cox models across candidate cut points, ensuring adequate group sizes.

To assess potential nonlinear associations between scar burden and risk, the number of LGE-positive segments was modeled as a continuous variable using restricted cubic splines (four degrees of freedom) within a Cox regression framework.

All statistical analyses were performed using Stata version 14.0 (StataCorp, College Station, TX, USA). A two-sided *p* value <0.05 was considered statistically significant.

## Results

The study cohort comprised 133 patients; 71 (53.4%) were women, with a median age of 64 years [interquartile range (IQR): 52–76 years]. Hypertension was the most common comorbidity (40.6%), followed by atrial fibrillation/flutter (22.3%) and diabetes mellitus (11.1%). Most patients (76.7%) were receiving angiotensin-converting enzyme inhibitors (ACEIs) or angiotensin receptor blockers (ARBs), including angiotensin receptor–neprilysin inhibitors (ARNIs). Beta-blockers were prescribed in 82.7% of the cohort.

Regarding guideline-directed medical therapy (GDMT) for heart failure—including ACEIs, ARBs, ARNIs, beta-blockers, sodium–glucose cotransporter 2 (SGLT2) inhibitors, and mineralocorticoid receptor antagonists—10 patients (7.5%) were not receiving any of these agents. Twenty-six patients (19.5%) were treated with one drug class, 38 (28.5%) with two classes, 34 (25.5%) with three classes, and 25 (18.8%) were receiving one agent from each of the aforementioned drug classes (Table 1).

**Table 1:** Patient characteristics and bivariate analysis for primary outcome.

Variable	All patients	Primary outcome		*p* Value*
	*n* = 133	Negative (*n* = 70)	Positive (*n* = 63)	
Age- yrs	64 ± 12	64 ± 16	63 ± 13	0.574
Male	62 (46.6)	29 (41.3)	33 (52.4)	0.205
Weight—kg	66.4 ± 0.8	65.4 ± 9.5	67.5 ± 9.6	0.222
BSA	1.7 ± 0.1	1.7 ± 0.1	1.7 ± 0.1	0.201
Hypertension	54 (40.6)	27 (38.6)	27 (42.9)	0.615
Diabetes mellitus	15 (11.3)	7 (10.0)	8 (12.7)	0.623
Atrial fibrillation/flutter	30 (22.3)	15 (21.4)	15 (23.8)	0.626
eGFR—mL/min/1,73 m^2^	77 ± 31	80 ± 29	73 ± 28	0.039
Medications				
Warfarin	16 (12.1)	7 (10.0)	9 (14.2)	0.448
DOAC	33 (24.8)	19 (27.1)	14 (22.2)	0.511
Aspirin	29 (21.8)	15 (21.4)	14 (22,2)	0.911
ARB	35 (26.3)	23 (32.9)	12 (19,1)	0.068
ACEi	35 (26.3)	17 (24.3)	18 (28,6)	0.575
ARNi	32 (24.1)	17 (24.3)	15 (23,8)	0.948
Beta Blocker	110 (82.7)	55 (78.6)	55 (87,3)	0.182
SGLT2i	32 (24.1)	16 (22.9)	16 (25,4)	0.732
MRA	62 (46.6)	29 (41.4)	33 (52,4)	0.205
Furosemide	29 (21.8)	12 (17.1)	17 (27,0)	0.169
Statin	55 (41.4)	31 (44.3)	24 (38,1)	0.468

Values are *n* (%), median (IQR) or mean ± SD. The *p* values refer to comparisons between positive versus negative combined endpoint and survivors versus no survivors.

* *p* values using chi-square, Student's *t*-test or Fisher exact test when appropriate.

### CMR derived parameters

The mean left ventricular ejection fraction (LVEF) was 43.3% ± 15%, and 39.8% of patients had an LVEF ≤40%. Left ventricular (LV) scar was detected in at least one LV segment in 97% of participants. The transmural pattern was the most frequent late gadolinium enhancement (LGE) pattern, observed in 75% of patients in at least one LV segment.

**Figure 1:**
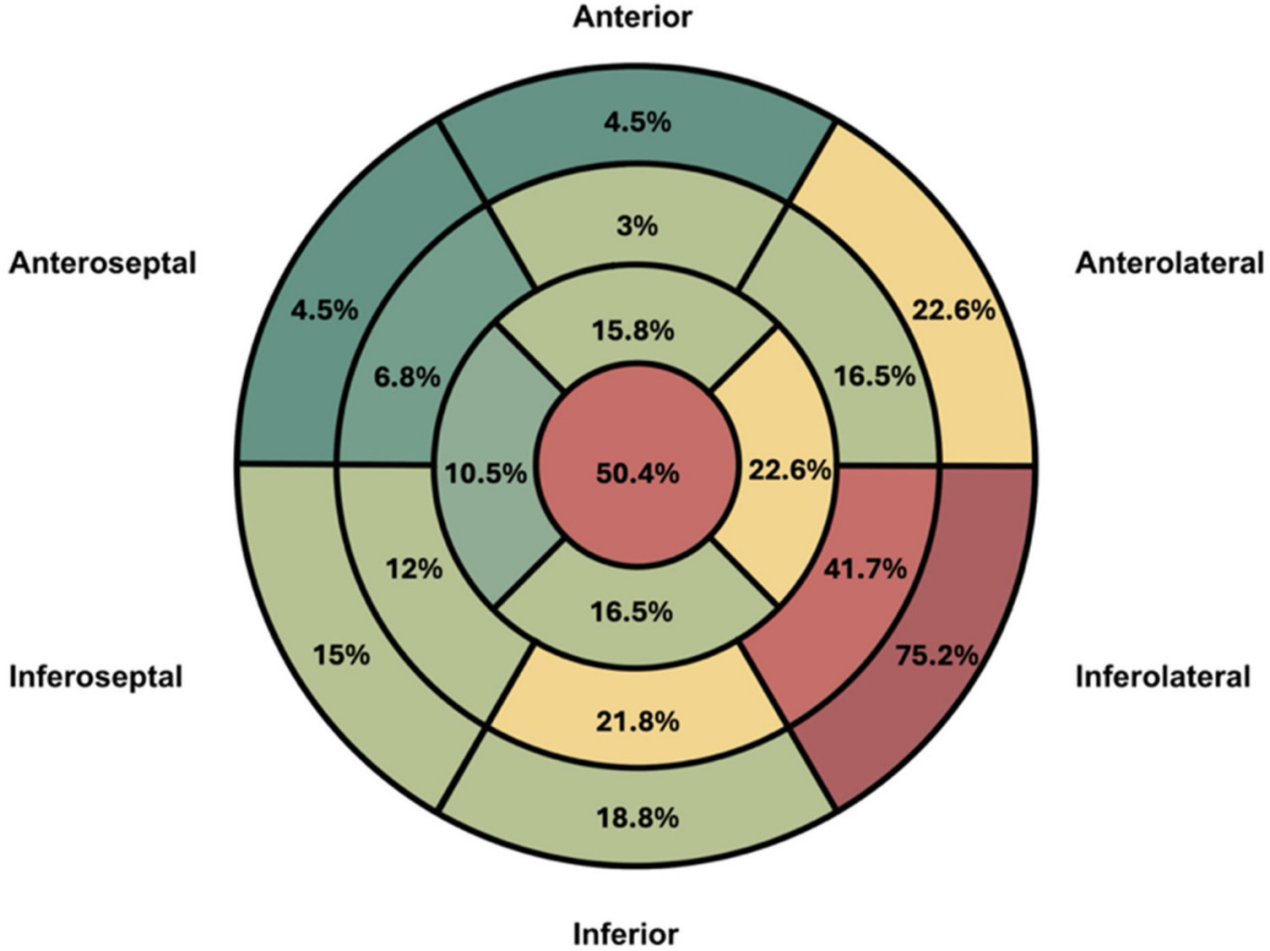
LV scar distribution. Number represents the percentage of patients with LGE in each LV segment. Red: ≥50% of patients. Yellow: 20%–50% of patients. Green: ≤20%. LV segmentation follows AHA 17-segment model. Adapted from: https://www.ahajournals.org/doi/10.1161/hc0402.102975.

The localization and distribution of LV scar are shown in Figure 1 and Supplementary Table S2, with the inferolateral wall and the apex being the most commonly affected regions. LV aneurysm and LV thrombus were identified in 21.1% and 12.8% of patients, respectively. Among patients with LV aneurysm, thrombus was present in 59% (Table 2).

**Table 2:** CMR characteristics and bivariate analysis for primary outcome.

Variable	Total	Primary combined outcome		*p* Value*
	*n* = 133	Without outcome *N* = 70	With outcome *N* = 63	
LVEF	43.3 ± 15.0	48.4 ± 14.3	37.7 ± 13.8	0.001
LVEF ≤ 40%—no, (%)	53 (39.8)	18 (25.7)	35 (55.6)	0.001
LV EDV—mL	178.5 ± 72	169 ± 73	198 ± 82	0.002
LV EDVi—ml/m^2^	101 ± 45	98 ± 32	107 ± 57	0.005
LV ESV—mL	98.5 ± 79	81.5 ± 53	122 ± 89	0.001
LV ESVi—mL/m^2^	57 ± 44	48.5 ± 28	69 ± 61	0.001
RVEF	52 ± 17.5	53 ± 13	46 ± 22	0.009
RVEF ≤ 51% (%)	63 (47.4)	26 (37.1)	37 (58.7)	0.012
RV EDV—mL	151 ± 50	147 ± 53	156 ± 55	0.196
RV EDVi BSA—mL/m^2^	89 ± 23	89 ± 25)	87 ± 28	0.331
RV ESV—ml	73 ± 45	70 ± 43	80 ± 61	0.035
RV ESVi BSA—mL/m^2^	44 ± 24	41 ± 20	46 ± 29	0.034
Scar	129 (97.0)	67 (95.7)	62 (98.4)	0.651
# of segments with scar	3.6 ± 2.4	3.17 ± 2.1	4.1 ± 2.6	0.027
Subendocardial	24 (18.1)	9 (12.9)	15 (23.8)	0.012
Mid-wall	71 (53.4)	36 (51.4)	35 (55.6)	0.633
Subepicardial	31 (23.3)	15 (21.4)	16 (25.4)	0.589
Transmural	95 (72.0)	51 (73.9)	44 (69.8)	0.603
Edema	28 (21.1)	14 (20.0)	14 (22.2)	0.753
LV aneurysm	28 (21.1)	14 (20.0)	14 (22.2)	0.753
LV thrombus	17 (12.8)	9 (12.9)	8 (12.7)	0.978

Values are *n* (%), or mean ± SD. The *p* values refer to comparisons between positive versus negative combined endpoint and survivors versus no survivors.

* *p* values using chi-square, Student's *t*-test or Fisher exact test when appropriate. LGE distribution categories were defined as including at least one LV segment with a category of LGE distribution.

### Outcomes

During a median follow-up of 554 days (IQR: 227–1,220 days), 63 patients (47.4%) experienced at least one component of the primary composite outcome. Specifically, 51 patients (38.3%) had at least one cardiovascular hospitalization, 20 (15.0%) died, 7 (5.2%) experienced an ischemic stroke, and 1 underwent heart transplantation. Notably, 42.1% of patients received an implantable cardioverter-defibrillator (ICD), with a median time from CMR to implantation of 9 days.

The multivariable analysis included age, subendocardial late gadolinium enhancement (LGE), right ventricular (RV) dysfunction [defined as right ventricular ejection fraction (RVEF) ≤ 51%], angiotensin receptor blocker use, male sex, LV systolic dysfunction (defined as LVEF ≤40%), renal dysfunction (defined as estimated glomerular filtration rate <60 mL/min/1.73 m^2^), and extensive LV scarring (defined as ≥6 segments with LGE).

LV end-diastolic volume (LVEDV) and LV end-systolic volume (LVESV) were strongly and inversely correlated with LVEF (*r* = −0.71, *p* < 0.001 for LVEDV; *r* = −0.83, *p* < 0.001 for LVESV). Similarly, RV end-systolic volume (RVESV) was inversely correlated with RVEF (*r* = −0.83, *p* < 0.001).

In the adjusted model, age [hazard ratio [HR] 1.49 per 10-year increase; 95% confidence interval [CI] 1.10–2.03; *p* = 0.01], subendocardial LGE (HR: 2.18; 95% CI: 1.18–4.03; *p* = 0.013), and extensive LV scarring (HR: 1.93; 95% CI: 1.06–3.52; *p* = 0.032) were independently associated with the primary outcome (Table 3).

**Table 3:** Multivariable cox proportional hazards model for the primary combined outcome (all-cause mortality, cardiovascular hospitalization, ischemic stroke, and heart transplantation) in patients with chronic chagas cardiomyopathy.

Variable	Hazard ratio (HR)	95% CI	*p*-value
Age (per 10-year increase)	1.49	1.10–2.03	0.010
Subendocardial LGE pattern	2.18	1.18–4.03	0.013
LGE ≥ 6 segments	1.93	1.06–3.52	0.032
RVEF ≤ 51%	1.43	0.79–2.59	0.240
ARB use	0.68	0.34–1.36	0.278
Male sex	0.80	0.47–1.36	0.414
LVEF ≤ 40%	1.04	0.55–1.96	0.910
eGFR < 60 mL/min	1.03	0.57–1.85	0.920

ARB, angiotensin receptor blocker; eGFR, estimated glomerular filtration rate; LVEF, Left ventricle ejection fraction; RVEF, right ventricle ejection fraction. Bold values indicate statistical significance (*p* < 0.05).

Model diagnostics supported the adequacy of the Cox proportional hazards model. Multicollinearity was low (variance inflation factors 1.09–1.46; mean VIF 1.24; condition number 19.57), indicating no clinically relevant collinearity among covariates. The proportional hazards assumption was not violated (Schoenfeld residuals global test *χ*^2^ = 7.65, df = 7; *p* = 0.364), supporting the interpretation of hazard ratios as constant over time. Only age remained statistically significantly associated with all-cause mortality in the adjusted analysis (Table 4).

**Table 4:** Multivariable cox proportional hazards model for all-cause mortality.

Variable	Hazard ratio (HR)	95% CI	*p*-value
Age (per 10-year increase)	1.89	1.03–3.49	0.041
Male sex	2.21	0.81–6.02	0.121
LGE ≥ 6 segments	1.76	0.59–5.21	0.307
RV dysfunction	1.70	0.51–5.69	0.392
LVEF ≤ 40%	1.44	0.37–5.60	0.602
ARB use	0.73	0.22–2.41	0.609
eGFR < 60 mL/min	1.19	0.41–3.46	0.750
Subendocardial LGE pattern	1.02	0.31–3.39	0.974

ARB, angiotensin receptor blocker; eGFR, estimated glomerular filtration rate; LVEF, left ventricle ejection fraction; RVEF, right ventricle ejection fraction. Bold values indicate statistical significance (*p* < 0.05).

**Figure 2:**
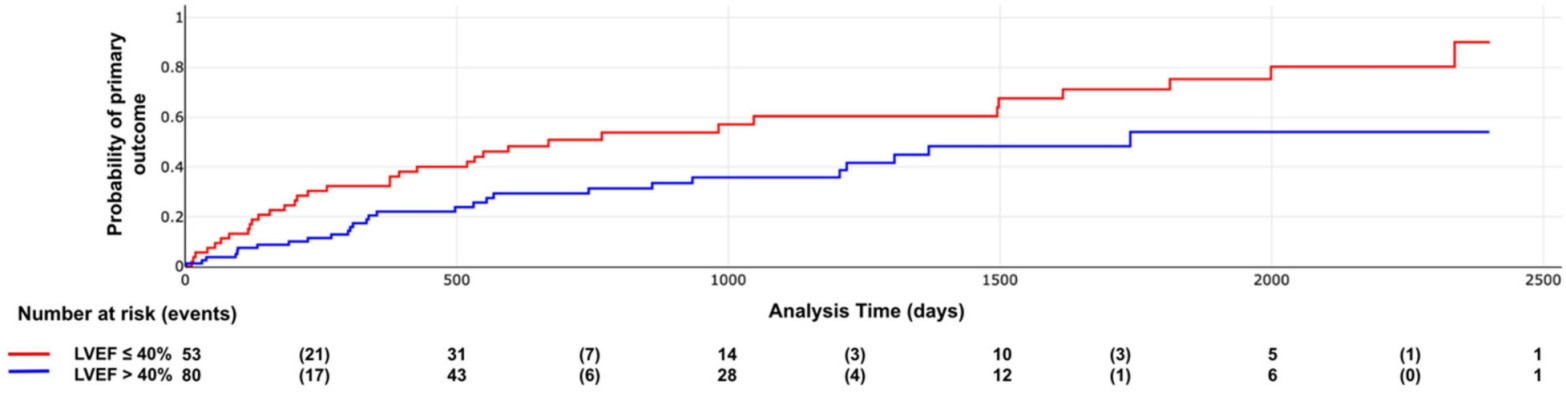
Primary outcome according to LVEF categories. Log rank X2: 7.04, *p* value = 0.007.

**Figure 3:**
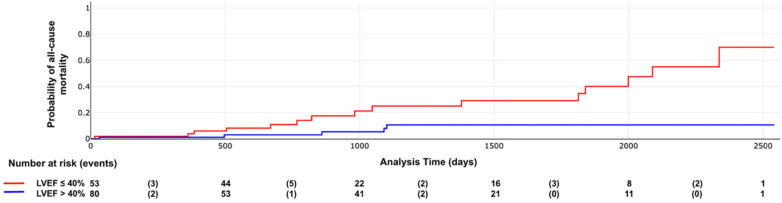
All-cause mortality according LVEF categories. Log rank X2: 9.38, *p* value = 0.002.

Kaplan–Meier curves demonstrated significant differences in survival distributions for both the primary outcome and all-cause mortality according to LVEF categories (log-rank *χ*^2^ = 7.04, *p* = 0.007; and *p* = 0.002, respectively; Figures 2, 3).

**Figure 4:**
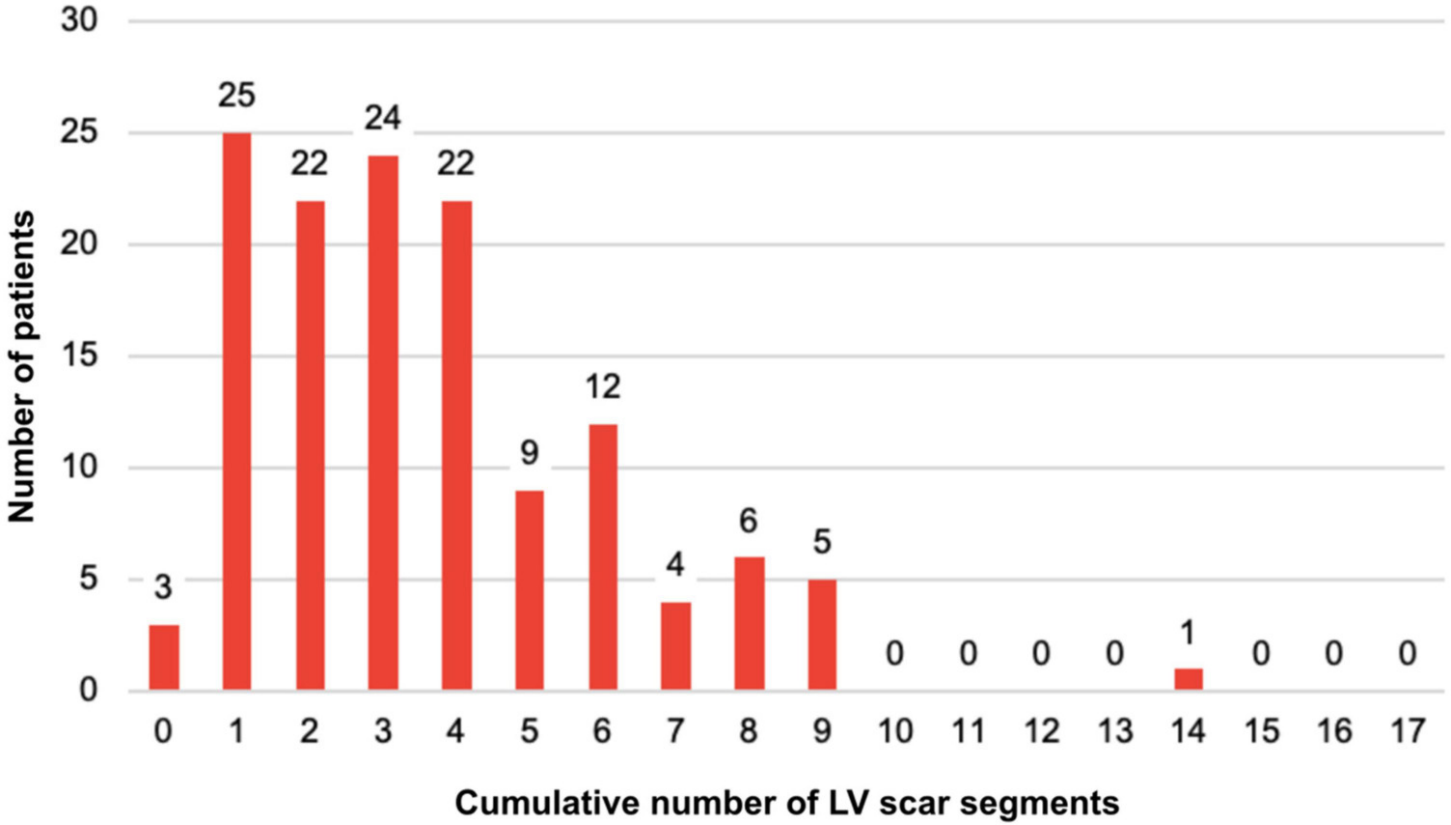
LV scar extent distribution.

**Figure 5:**
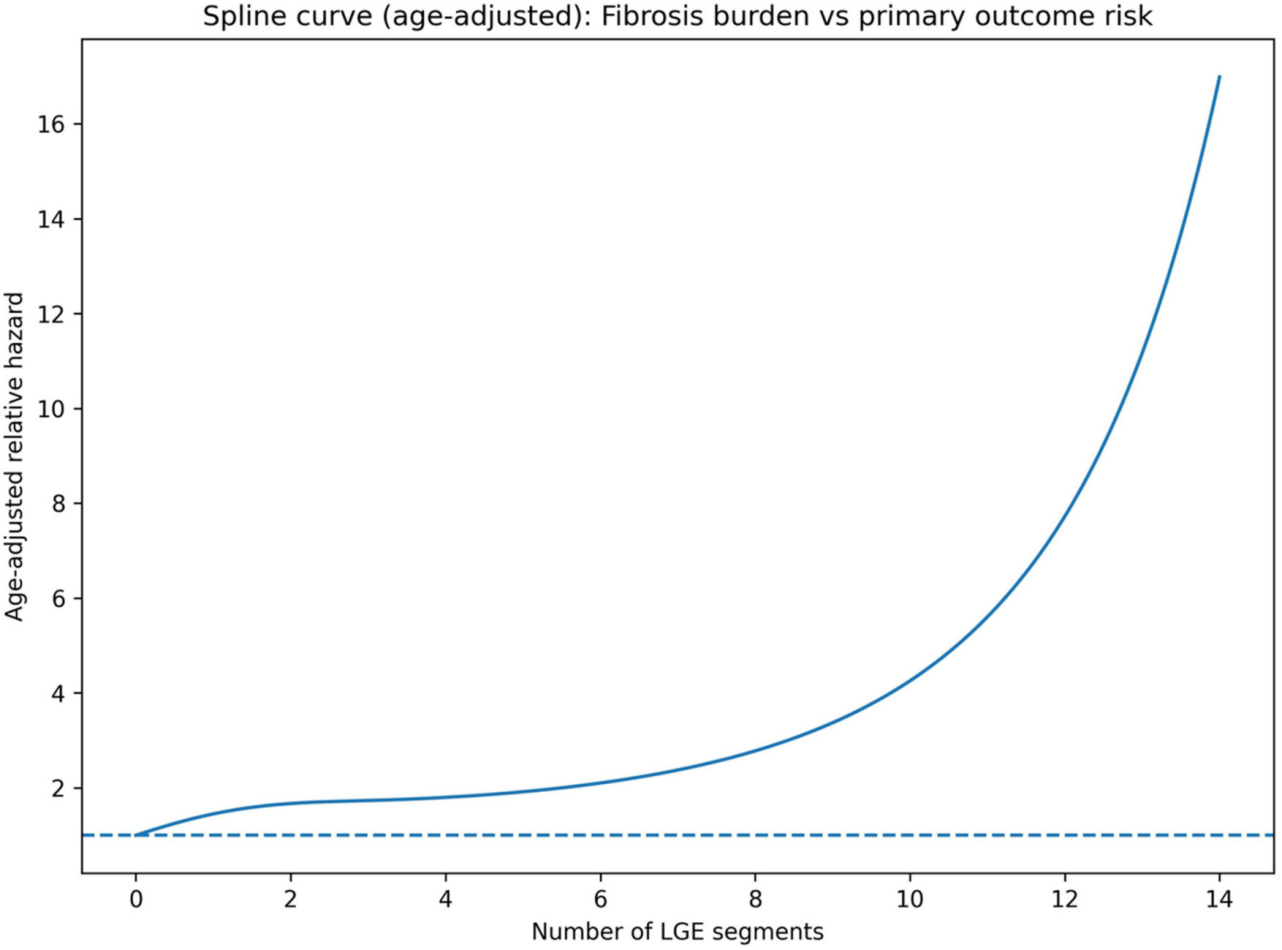
Age-adjusted restricted cubic spline analysis of myocardial fibrosis burden and risk of the primary outcome.

LV scar extent was not normally distributed, with a median of 3 segments (IQR: 3) (Figure 4). Restricted cubic spline modeling demonstrated a nonlinear association between the number of myocardial segments with LGE and the hazard of the primary composite outcome after adjustment for age (Figure 5). Risk remained relatively stable at low scar burden, began to increase beyond approximately 4–6 segments, and rose steeply at higher degrees of fibrosis.

**Figure 6:**
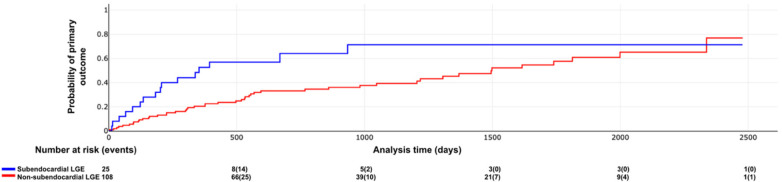
Primary outcome according subendocardial LGE. Log rank X2 = 6.77, *p* value 0.009.

**Figure 7:**
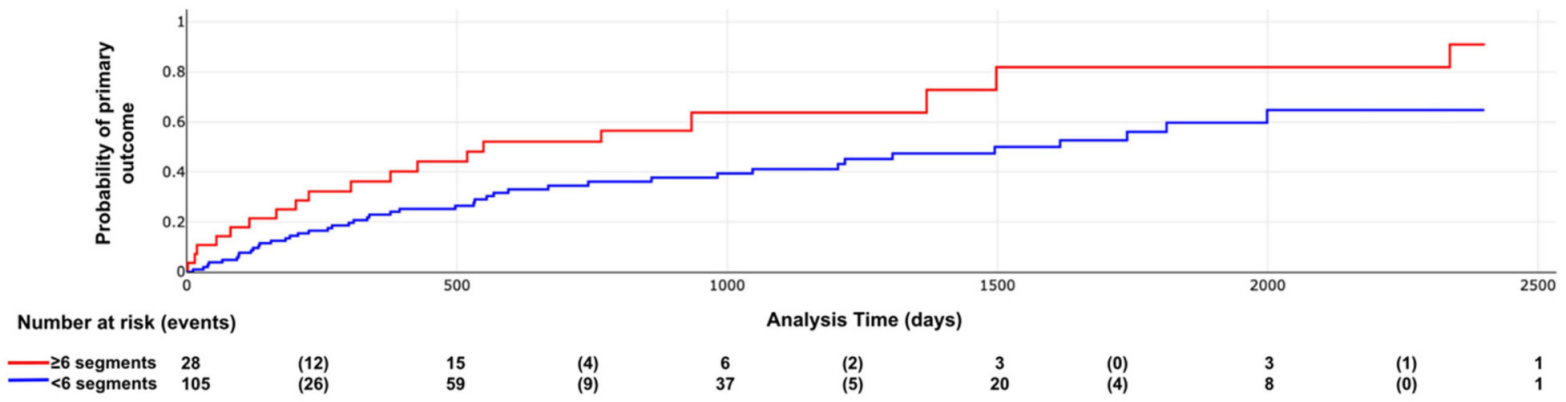
Primary outcome according LV scar extent. Log rank X2 = 5.25, *p* value = 0.021.

**Figure 8:**
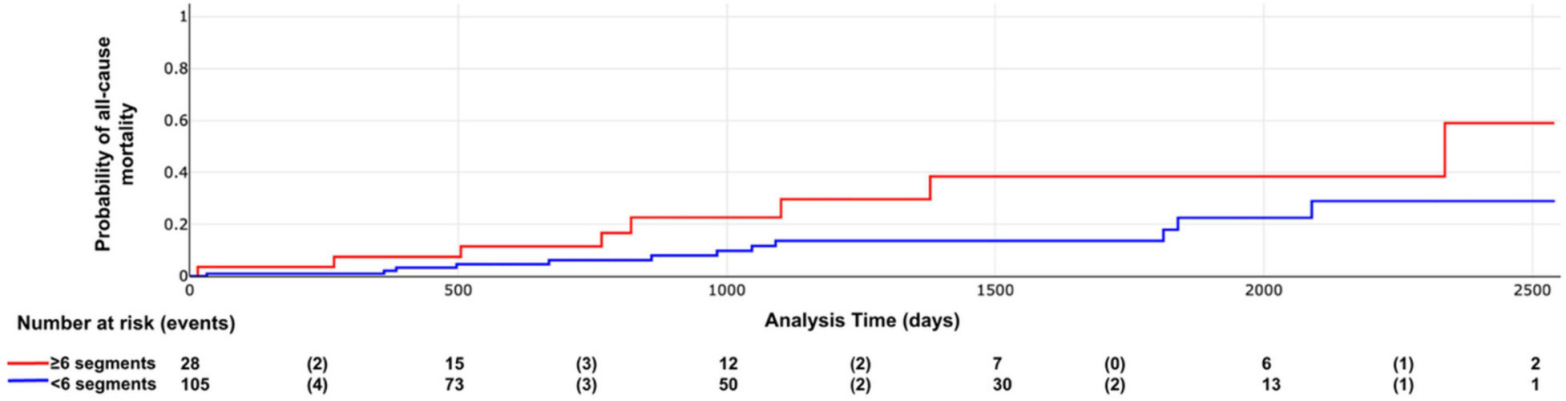
All-cause mortality according to LV scar extent. Log rank X2 = 3.33, *p* value = 0.067.

Kaplan–Meier curves illustrating the association between LV scar extent and pattern with outcomes are shown in Figures 6–8. The subendocardial LGE pattern was significantly associated with the primary outcome (log-rank *χ*^2^ = 6.77; *p* = 0.009; Figure 6). When analyzed categorically using a cutoff of ≥6 LGE-positive segments, LV scar extent was significantly associated with the primary outcome (log-rank *χ*^2^ = 5.25; *p* = 0.021; Figure 7), but not with all-cause mortality (log-rank *χ*^2^ = 3.33; *p* = 0.067; Figure 8).

**Figure 9:**
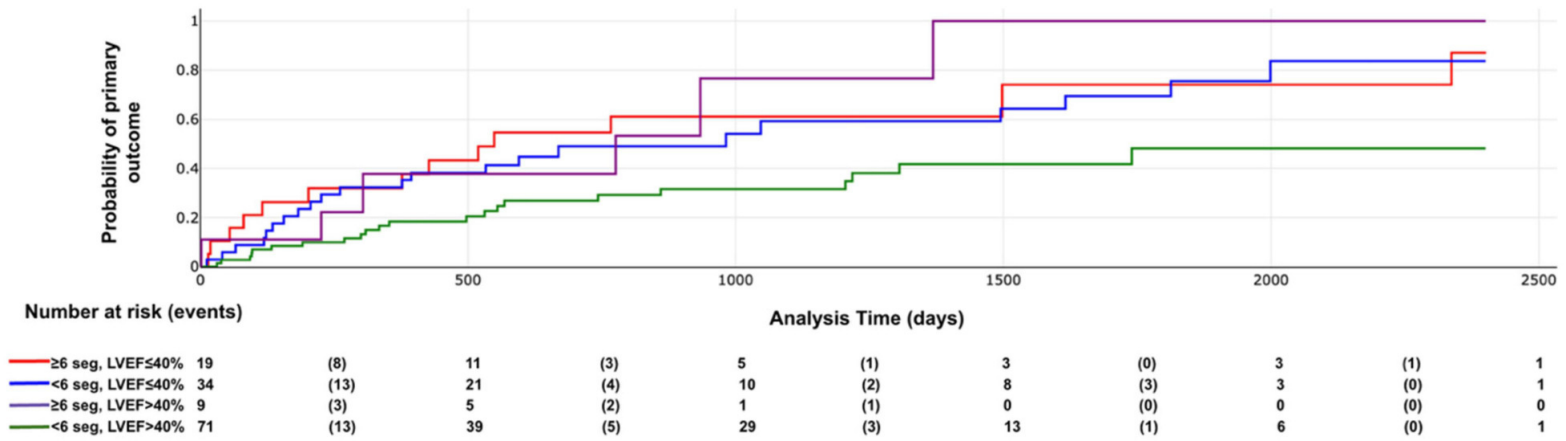
Primary outcome according LV scar extent and LVEF categories. Log rank X2 = 11.51, *p* value = 0.009.

**Figure 10:**
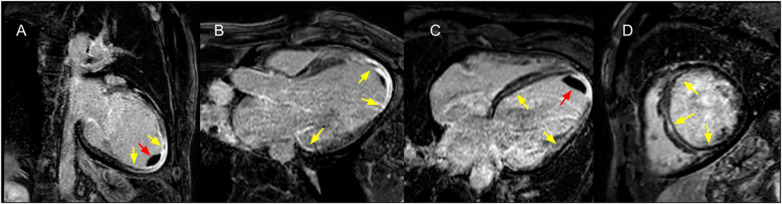
Cardiac magnetic resonance (CMR) of a 62-year-old male patient with chagas cardiomyopathy. Left ventricular severe dilation, with diffuse LV scar (depicted with late gadolinium enhancement) in **(A)** 2 chamber view, **(B)** LV outflow tract view, **(C)** 4 chamber view and **(D)** short axis view at mid-level, affecting 8 LV segments (yellow arrows) and apical thrombus (red arrows). Baseline LVEF was 30%.

**Figure 11:**
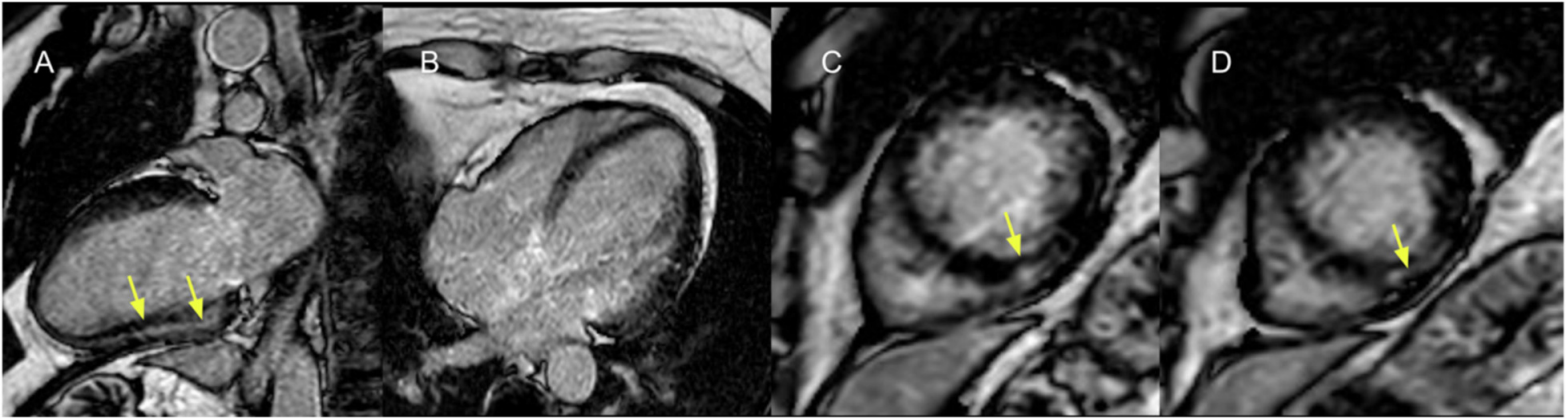
Cardiac magnetic resonance (CMR) of a 67-year-old female patient with chagas cardiomyopathy. Left ventricular moderate dilation with localized subepicardial LV scar (depicted with late gadolinium enhancement) in **(A)** 2-chamber view, **(B)** 4-chamber view, and **(C,D)** short-axis view at mid-level, affecting 3 LV segments (yellow arrows). Baseline LVEF was 38%.

When patients were stratified into four groups according to LV systolic function (LVEF ≤40% vs. >40%) and LV scar extent (≥6 vs. <6 LGE-positive segments), a significant difference in the incidence of the primary outcome was observed (log-rank *χ*^2^ = 11.51; *p* = 0.009; Figure 9; representative examples in Figures 10, 11). Among patients with LVEF >40%, extensive LV scarring (≥6 segments) remained independently associated with the primary outcome after adjustment for age (HR: 2.73; 95% CI: 1.08–6.90; *p* = 0.034).

## Discussion

In this cohort of patients with CC, the incidence of major adverse cardiac events was high, affecting nearly half of the population over a mean follow-up of 1.5 years. CMR–derived parameters demonstrated significant prognostic value. In particular, the pattern and extent of LV scar—quantified by the number of segments with LGE—were independently associated with the primary composite outcome, with a threshold of ≥6 affected segments identifying patients at increased risk. Importantly, even among individuals with LVEF >40%, greater scar burden remained a strong predictor of adverse events. In contrast, other structural abnormalities, RV dysfunction, LV aneurysm, and intracardiac thrombus, were not independently associated with the primary outcome (Central Illustration).

**Central Illustration:**
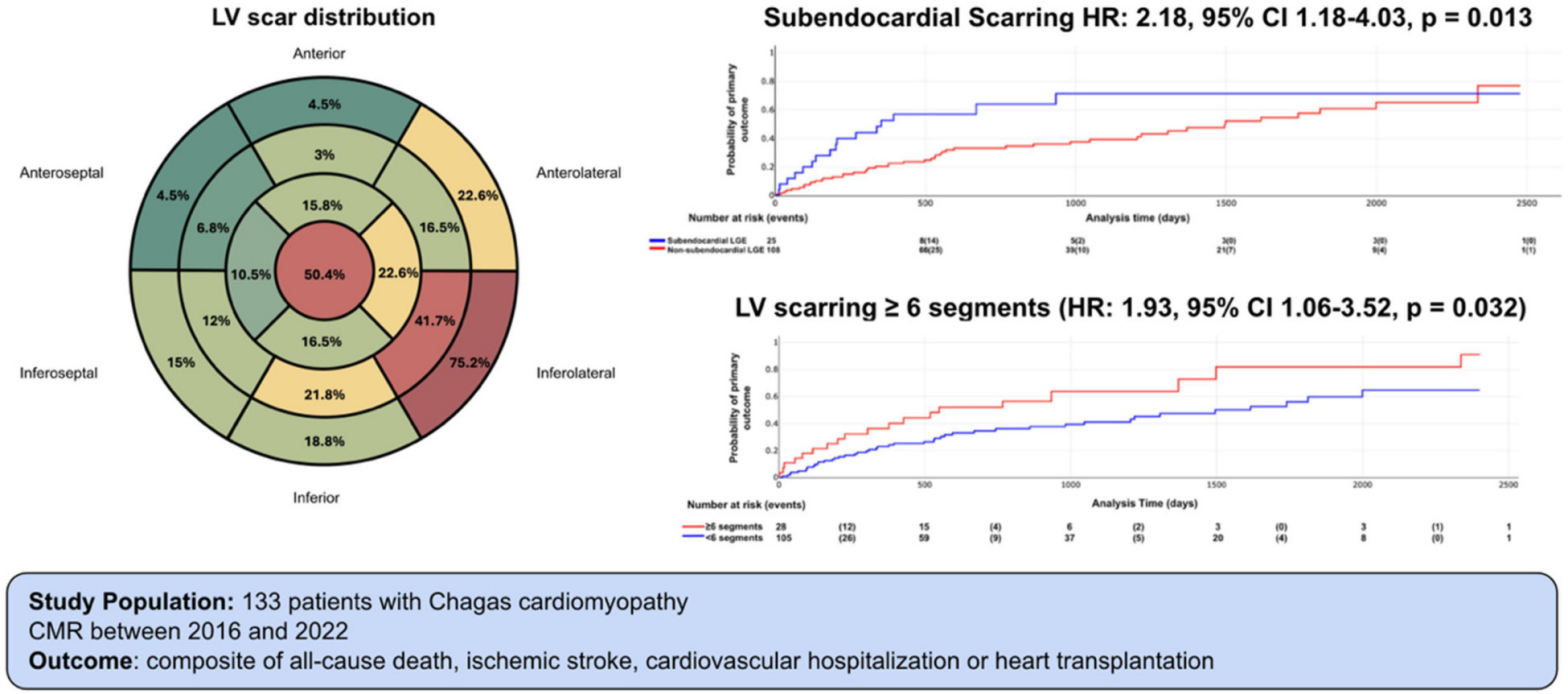
LV scar extent and pattern are predictors of adverse clinical outcomes in patients with Chagas cardiomyopathy.

Although the elevated risk of cardiovascular complications in CC is well established, the interpretation and clinical integration of CMR findings for risk stratification remain areas of ongoing investigation (18). This challenge is particularly relevant in endemic regions and low- and middle-income countries, where access to advanced imaging techniques is limited (14). In this context, our findings provide clinically relevant data to refine prognostic assessment.

First, although reduced LVEF has consistently been reported as a major prognostic marker in CC, it was not independently associated with the primary outcome in our adjusted analysis (5, 12, 19–23). While early detection of LV systolic dysfunction remains essential for therapeutic decision-making, our results suggest that reliance on LVEF alone may underestimate risk in certain patients. Notably, the incidence of adverse outcomes remained substantial among patients with relatively preserved LVEF.

Although RV systolic dysfunction was associated with the composite outcome in univariable analysis, this association did not persist after multivariable adjustment. Prior studies have demonstrated a high prevalence of RV dysfunction in CC. Moreira et al. reported a 37% prevalence of RV systolic dysfunction assessed by CMR, which was associated with LV systolic dysfunction and atrial fibrillation (24). Other investigations using different imaging modalities have identified RV dysfunction as an independent predictor of adverse outcomes and reduced exercise capacity in CC (25, 26). The lack of independent association in our cohort may reflect collinearity with LV dysfunction or limited statistical power.

Second, we evaluated the prognostic role of LV scar characterization. Myocardial fibrosis in CC exhibits distinctive features compared with other cardiomyopathies, and several mechanisms have been proposed to explain its distribution. The preferential involvement of apical and basal inferolateral segments may be related to episodes of ischemia during inflammatory phases, particularly in territories susceptible to coronary steal (distal left anterior descending and left circumflex arteries). This phenomenon has been attributed to microvascular dysfunction secondary to inflammatory-mediated vasodilation of proximal vascular beds (27).

Microvascular ischemia may partially explain the occurrence of subendocardial scarring and its independent prognostic significance in our population. Subendocardial LGE has also been investigated as a prognostic marker in other cardiomyopathies, including hypertrophic cardiomyopathy and autoimmune-related myocardial disease (28, 29).

The prevalence of LV scar in our cohort was high (97%), with distribution patterns similar to previous reports, predominantly involving the inferolateral basal segments and the apex, despite the heterogeneous nature of CC (18, 30). Apical involvement was particularly frequent, affecting nearly half of the patients. Transmural and mid-wall LGE patterns were most common, consistent with prior observations (31).

LV scar presence and extent have consistently been associated with adverse outcomes in CC (19, 21, 32–34). Volpe et al. reported LV scar (present in 71.4% of patients) as a predictor of cardiovascular death or sustained ventricular tachycardia (HR: 1.06; 95% CI: 1.01–1.12; *p* = 0.045) (21). Similarly, Senra et al. demonstrated that myocardial fibrosis predicted a composite of all-cause mortality, heart transplantation, appropriate implantable cardioverter-defibrillator (ICD) therapy, antitachycardia pacing, or aborted sudden cardiac death (19). A recent meta-analysis further confirmed the association between LGE and mortality as well as composite adverse outcomes in CC (18).

In our study, LV scar burden—quantified as the cumulative number of LGE-positive segments—was moderately correlated with LVEF, LV end-diastolic volume, and LV end-systolic volume. A threshold of ≥6 segments provided meaningful risk discrimination. Quantification of LGE by segmental burden has previously demonstrated prognostic value in ischemic and non-ischemic cardiomyopathies (35, 36). Although advanced techniques such as T1 mapping and extracellular volume (ECV) quantification have shown promise in CC, their availability remains limited in many endemic settings (37).

Importantly, patients with extensive LV scar but preserved LVEF had a prognosis comparable to those with LVEF ≤40%. This finding underscores the incremental prognostic value of scar assessment beyond systolic function alone. Consistent with this observation, a large meta-analysis involving more than 29,000 patients with non-ischemic dilated cardiomyopathy demonstrated that LGE presence and extent were superior to LVEF for risk prediction (38).

LV scar burden has also been associated with ventricular arrhythmias in CC and other cardiomyopathies (6, 39–41). In our cohort, 42.1% of patients underwent early ICD implantation, which may have mitigated arrhythmic mortality. This could partially explain why LV scar extent was not independently associated with all-cause mortality in adjusted analyses.

From a clinical standpoint, LGE-based scar assessment may meaningfully refine risk stratification in CC. Early identification of patients with extensive fibrosis—even in the setting of preserved LVEF—could support closer follow-up, optimization of guideline-directed medical therapy, and timely consideration of device-based interventions.

## Limitations

Our results should be interpreted within the appropriate context: the population presented in an advanced stage of CC, with no individuals in acute or indeterminate stages. This limitation may not fully represent the overall population or encompass the entire natural history of patients with CC. CMR was indicated for various clinical implications, particularly for the identification of left ventricular (LV) thrombus or prior to implantable cardioverter-defibrillator (ICD) implantation, where it aids in identifying LV scar zones that may contribute to arrhythmogenic substrate formation.

This study has several limitations. First, due to its retrospective design, causality cannot be inferred from the findings. Additionally, the indication for performing CMR imaging may have introduced a selection bias toward patients with more severe forms of the disease. The absence of serial imaging limits the ability to generalize findings and precludes evaluation of dynamic changes in myocardial fibrosis and cardiac remodeling over time. Clinical outcomes were obtained from medical registries and self-reported data from patients or their relatives, which may be subject to reporting bias. However, all patients were either contacted directly or clinically assessed, minimizing the likelihood of significant missing outcome data. Quantitative analysis of diffuse LV scarring using T1 mapping was not performed, as the necessary techniques were not available during the study period, as well as LV longitudinal strain. Data on ventricular arrhythmias and implantable cardioverter-defibrillator (ICD) therapies were also not collected.

## Conclusion

LV scar pattern (subendocardial) and extension (LGE ≥6 segments) are independent prognostic factors for adverse outcomes in patients with CC. LV scar extent predicts adverse outcomes, even in patients with LVEF >40%. Prospective data are needed to clarify the role of scar analysis and CMR parameters in risk stratification for CC patients.

## Data Availability

The raw data supporting the conclusions of this article will be made available by the authors, without undue reservation.
