# Supplementary material for: Cardiac magnetic resonance predictors of adverse outcomes in Chagas cardiomyopathy
Source: Front Cardiovasc Med. 2026 Mar 17;13:1758594. doi: 10.3389/fcvm.2026.1758594 (PMC13035524; doi:10.3389/fcvm.2026.1758594)
Supplement: Supplementary file 1 [file Supplementaryfile1.docx]

**Table 1. Cardiovascular hospitalization**

| **Category** | **Definition** | **Key Criteria / Notes** | **Examples (if applicable)** |
| --- | --- | --- | --- |
| **Cardiovascular hospitalization** | An admission to the hospital with a **defined cardiovascular cause**. | Must have sufficient documentation to adjudicate the cause as cardiovascular. It is **not the default** category when the cause is unclear. | Acute myocardial infarction; arrhythmia or conduction disturbance; cardiogenic shock; cardiovascular device failure; cardiovascular haemorrhage (including tamponade); cardiovascular infection; cardiovascular procedure-related complications; heart failure (left or right ventricular dysfunction); peripheral arterial disease; thromboembolism; stroke; sudden cardiac death. |
| **Non-cardiovascular hospitalization** | An admission to the hospital with a **defined cause that can be adjudicated as non-cardiovascular**. | Requires adequate information to clearly determine that the primary cause is not cardiovascular. Should **not** be assigned if the cause cannot be adjudicated. | Not specified in the statement (any clearly non-cardiovascular medical or surgical cause, when well documented). |
| **Unknown cause hospitalization** | An admission where the cause **is not known, not clearly documented, or cannot be adjudicated** as cardiovascular or non-cardiovascular. | Many hospitalizations have insufficient documentation. In these cases, they **must not be labeled** as cardiovascular or non-cardiovascular. The consensus recommends **pre-specifying** how these events will be classified and analyzed in study protocols. | Hospitalizations with unclear, missing, or insufficient diagnostic information. |

Anexo 2. Guion telefónico para consentimiento informado.

Cordial saludo, mi nombre es ______________, médico investigador de laCardio. Nos encontramos realizando una investigación en pacientes con enfermedad de Chagas que fueron atendidos en nuestra institución, a quienes se les realizó un examen de resonancia magnética de corazón entre los años 2015 y 2022. Este estudio incluye un cuestionario con una serie de preguntas sobre su historial médico (o de su familiar), por lo cual, es importante conocer si usted estaría dispuesto a continuar con la llamada, y si acepta participar, responder una encuesta al final de la llamada.

A continuación, me permito informar de qué trata el estudio y la forma en la que usted podría participar. En caso de tener alguna duda por favor pregúnteme para poder aclararla. Haga todas las preguntas que usted crea convenientes durante cualquier momento antes de informarnos su decisión. Nosotros responderemos todas las preguntas con información verdadera y clara.

¿De qué trata el estudio?: Queremos realizar una investigación que nos permita analizar su examen de resonancia realizada en laCardio, conocer su estado de salud actual y saber si algunas características de ese examen pueden estar relacionadas con su estado de salud. Esto nos ayudará a conocer más sobre la enfermedad de Chagas.

Procedimientos del estudio: Si usted quiere participar voluntariamente en este estudio, durante esta llamada debe responderme unas preguntas muy sencillas sobre su estado de salud actual y en caso de haber asistido a laCardio para hospitalizarse, nos autorizaría a revisar su historia clínica para confirmar datos en caso de requerirse. Toda la información que se envíe a este laboratorio estará anonimizada, es decir, no tendrá ningún dato que pueda identificarlo. Después analizaremos esa información junto con las preguntas que usted responda. Las únicas personas que sabrán que usted participó en el estudio somos los miembros del equipo de investigación. Nosotros no divulgaremos ninguna información sobre usted. Su nombre no aparecerá ni en los resultados ni en bases de datos.

Usted no recibirá ninguna remuneración económica ni de ningún tipo por participar en este estudio y tampoco tendrá que pagar ningún dinero por el análisis que se haga a su examen de resonancia. Tampoco se le realizarán otros procedimientos médicos adicionales con base en sus respuestas.

¿Usted desearía participar en este estudio?. Si la respuesta es si, continuar con las siguientes preguntas:

1. ¿Fue usted (o su familiar) hospitalizado a causa de su corazón (arritmias, dolor torácico, edemas, ahogo) luego de la fecha de realización de la resonancia cardíaca?

a. No __.

b. Si __. ¿Recuerda la fecha? ______________________

c. En caso de responder si, ¿recibió un diagnóstico de falla cardíaca descompensada, fibrilación auricular, taquicardia ventricular o fibrilación ventricular?

2. ¿Fue usted (o su familiar) hospitalizado por síntomas como adormecimiento, entumecimiento, hormigueo o debilidad en una mitad de la cara, brazo o pierna (especialmente en un lado del cuerpo) o por problemas para hablar luego de la fecha de realización de la resonancia cardíaca?

a. No __.

b. Si __. ¿Recuerda la fecha? ______________________

c. En caso de responder si, ¿recibió un diagnóstico de ataque cerebrovascular o accidente isquémico transitorio cerebral?

3. ¿Ha recibido usted (o su familiar) un trasplante cardíaco?

a. No __.

b. Si __. ¿Recuerda la fecha? ______________________

4. ¿Le ha sido implantado a usted (o su familiar) un dispositivo cardíaco de estimulación como marcapasos, cardiodesfibrilador o resincronizador?

a. No __.

b. Si __. ¿Recuerda la fecha? ______________________

Translation to english:

Annex 2. Telephone Script for Informed Consent

Kind regards, my name is ______________, a physician and researcher at LaCardio. We are conducting a research study on patients with Chagas disease who were treated at our institution and underwent a cardiac MRI between the years 2015 and 2022. This study includes a questionnaire with a series of questions about your (or your relative’s) medical history. Therefore, it is important to know if you are willing to continue with the call and, if you agree to participate, to answer a survey at the end of the call.

I will now explain what the study is about and how you could participate. If you have any questions, please ask so I can clarify them. Feel free to ask any questions you think are appropriate at any time before making your decision. We will answer all questions with truthful and clear information.

What is the study about?

We want to conduct research that will allow us to analyze your MRI performed at LaCardio, learn about your current health status, and determine whether any features of that MRI are related to your current condition. This will help us better understand Chagas disease.

Study procedures:

If you voluntarily choose to participate in this study, during this call you will answer a few very simple questions about your current health status. If you were hospitalized at LaCardio, you would authorize us to review your medical records to confirm some information if necessary. All information sent to the lab will be anonymized, meaning it will contain no identifying information. We will then analyze that data along with your responses. The only people who will know you participated in the study are the members of the research team. We will not disclose any information about you. Your name will not appear in the results or in any databases.

You will not receive any financial or other compensation for participating in this study, and you will not have to pay anything for the analysis of your MRI. No additional medical procedures will be performed based on your responses.

Would you like to participate in this study?

If the answer is yes, continue with the following questions:

Were you (or your relative) hospitalized due to heart-related issues (arrhythmias, chest pain, edema, shortness of breath) after the date of the cardiac MRI?

a. No __

b. Yes __. Do you remember the date? ______________________

c. If yes, were you diagnosed with decompensated heart failure, atrial fibrillation, ventricular tachycardia, or ventricular fibrillation?

Were you (or your relative) hospitalized for symptoms such as numbness, tingling, or weakness in one side of the face, arm, or leg (especially one side of the body), or for speech difficulties after the date of the cardiac MRI?

a. No __

b. Yes __. Do you remember the date? ______________________

c. If yes, were you diagnosed with a stroke or transient ischemic attack (TIA)?

Have you (or your relative) received a heart transplant?

a. No __

b. Yes __. Do you remember the date? ______________________

Have you (or your relative) had a cardiac device implanted, such as a pacemaker, defibrillator, or resynchronizer?

a. No __

b. Yes __. Do you remember the date? ______________________

Supplemental file 2

|  | **LGE pattern** | | | | |
| --- | --- | --- | --- | --- | --- |
| **LV segment** | **Subendocardial (%)** | **Mid wall (%)** | **Subepicardial (%)** | **Transmural (%)** | **Without LGE (%)** |
| **Segment 1** | 2 (1,5) | 3 (2,3) | 1 (0,8) | 0 | 127 (95,5) |
| **Segment 2** | 1 (0,8) | 29 (21,8) | 0 | 3 (2,3) | 100 (75,2) |
| **Segment 3** | 0 | 17 (12,8) | 1 (0,8) | 2 (1,5) | 113 (85,0) |
| **Segment 4** | 2 (1,5) | 10 (7,5) | 2 (1,5) | 11 (8,3) | 108 (81,2) |
| **Segment 5** | 11 (8,3) | 17 (12,8) | 14 (10,5) | 58 (43,6) | 33 (24,8) |
| **Segment 6** | 4 (3,0) | 5 (3,8) | 6 (4,5) | 15 (11,3) | 103 (77,4) |
| **Segment 7** | 2 (1,5) | 2 (1,5) | 0 | 0 | 129 (97,0) |
| **Segment 8** | 1 (0,9) | 6 (4,5) | 0 | 2 (1,5) | 124 (93,2) |
| **Segment 9** | 2 (1,5) | 13 (9,8) | 0 | 1 (0,8) | 117 (88,0) |
| **Segment 10** | 1 (0,8) | 10 (7,5) | 7 (5,3) | 11 (8,3) | 104 (78,2) |
| **Segment 11** | 2 (1,5) | 17 (12,3) | 5 (3,8) | 31 (23,5) | 77 (58,3) |
| **Segment 12** | 1 (0,8) | 5 (3,8) | 1 (0,8) | 15 (11,3) | 111 (83,5) |
| **Segment 13** | 2 (1,5) | 1 (0,8) | 2 (1,5) | 16 (12,0) | 112 (84,2) |
| **Segment 14** | 1 (0,8) | 0 | 0 | 13 (9,8) | 119 (89,5) |
| **Segment 15** | 1 (0,8) | 3 (2,3) | 1 (0,8) | 17 (12,8) | 111 (83,5) |
| **Segment 16** | 2 (1,5) | 1 (0,8) | 3 (2,3) | 23 (18,1) | 103 (77,4) |
| **Segment 17** | 3 (2,3) | 0 | 0 | 64 (48,1) | 66 (49,6) |
